# A digital nervous system aiming toward personalized IoT healthcare

**DOI:** 10.1038/s41598-021-87177-z

**Published:** 2021-04-08

**Authors:** Astrid Armgarth, Sandra Pantzare, Patrik Arven, Roman Lassnig, Hiroaki Jinno, Erik O. Gabrielsson, Yonatan Kifle, Dennis Cherian, Theresia Arbring Sjöström, Gautier Berthou, Jim Dowling, Takao Someya, J. Jacob Wikner, Göran Gustafsson, Daniel T. Simon, Magnus Berggren

**Affiliations:** 1 Laboratory of Organic Electronics, Department of Science and Technology, Linköping University, 601 74 Norrköping, Sweden; 2 Printed Electronics, RISE Research Institute of Sweden AB, 602 21 Norrköping, Sweden; 3 Electrical Engineering, J2 Holding AB, 59533 Mjölby, Sweden; 4 RIKEN Center for Emergent Matter Science, 2-1 Hirosawa, Wako, Saitama 351-0198 Japan; 5 Electrical and Electronic Engineering and Information Systems, University of Tokyo, 7-3-1 Hongo, Bunkyo-ku, Tokyo, 113-8656 Japan; 6 Department of Electrical Engineering, Linköping University, 581 83 Linköping, Sweden; 7 RISE SICS, Research Institutes of Sweden AB, Kista, Sweden; 8 Department of Software and Computer Systems, School of ICT, KTH Royal Institute of Technology, Stockholm, Sweden

**Keywords:** Electrical and electronic engineering, Actuators, Sensors and biosensors, Health care, Mathematics and computing

## Abstract

Body area networks (BANs), cloud computing, and machine learning are platforms that can potentially enable advanced healthcare outside the hospital. By applying distributed sensors and drug delivery devices on/in our body and connecting to such communication and decision-making technology, a system for remote diagnostics and therapy is achieved with additional autoregulation capabilities. Challenges with such autarchic on-body healthcare schemes relate to integrity and safety, and interfacing and transduction of electronic signals into biochemical signals, and vice versa. Here, we report a BAN, comprising flexible on-body organic bioelectronic sensors and actuators utilizing two parallel pathways for communication and decision-making. Data, recorded from strain sensors detecting body motion, are both securely transferred to the cloud for machine learning and improved decision-making, and sent through the body using a secure body-coupled communication protocol to auto-actuate delivery of neurotransmitters, all within seconds. We conclude that both highly stable and accurate sensing—from multiple sensors—are needed to enable robust decision making and limit the frequency of retraining. The holistic platform resembles the self-regulatory properties of the nervous system, i.e., the ability to sense, communicate, decide, and react accordingly, thus operating as a digital nervous system.

## Introduction

The internet-of-things (IoT) paradigm is currently being implemented, in the widest sense, in society and industry, and has become a reality thanks to synergetic progress in electronics, computer science, communication technology, informatics, as well as social and medical sciences^1^. On the hardware side, IoT includes sensors and actuators, placed on and inside items, systems, animals, and humans, that together form wired or wireless networks connected to information technology infrastructure. On the software side, various protocols^2^ assist the accumulation of sensory data, for cloud/cloudlet/fog computation^3,4^ and decision-making, which further instruct various actuations and services. For humans, IoT solutions are already in use and heavily explored to support and assist us in daily life, for safety, entertainment, and in healthcare^5^. For personalized and participatory medicine there is a particularly promising pathway for advanced healthcare operated outside the hospital. Here, monitoring and regulation/actuation of the ambience, as well as the parameters and functions expressed on the skin and inside our body, defines an array of crucial factors to initiate or fine-tune therapy and other healthcare solutions.

Over the past several years, an array of tools and microfabricated technologies have been developed for monitoring health status and providing personalized/remote therapy^6–10^. In parallel, machine learning algorithms have been developed for early disease detection, as well as enhanced patient care and utilization of community services^11^. Finally, body-area networks (BANs)^12^ of sensors and therapeutic devices have been proposed as a solution to the fundamental challenges of security^13^ and patient privacy. BANs can provide a highly localized “cloudlet” limited to the body connecting bioelectronic nodes on/in the body with a personal digital assistant (e.g., mobile phone) and thereby limiting—and securing—the connection to cloud-based machine learning algorithms for optimized personalized medicine^14–16^. To date, healthcare-focused BANs and the vast majority of e-health wearables (e.g., FitBit, Apple Watch) have been focused on monitoring health status parameters via one or more sensors. More recent developments in healthcare-focused BANs have included either machine learning for optimized signal processing and health-status recognition^14,16–19^ or included drug-delivery components for more closed-loop care^20^ (similar to other closed-loop systems such as the “artificial pancreas” for diabetes treatment^21^). However, the three advances in healthcare technologies detailed above—(i) bioelectronic sensors/therapeutics, (ii) machine learning algorithms for personalized medicine, and (iii) healthcare BANs—have not been integrated into a unified, secure, “smart” e-health system.

**Figure 1:**
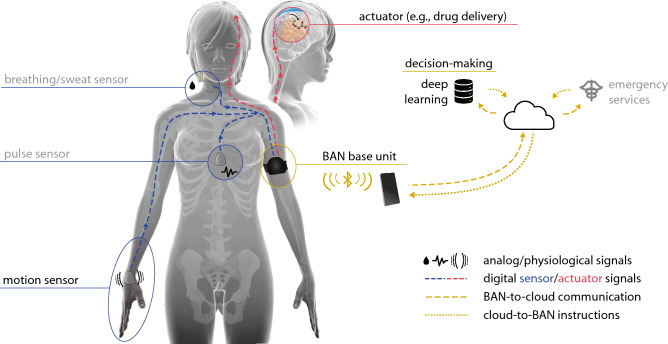
The digital nervous system (DNS), a smart healthcare body-area network (BAN). Separate nodes on the human body work in unison to sense and communicate health parameters and actuate medical treatments. Sensor nodes (e.g., breathing/sweat, pulse, motion) convert physiological information into digital signals which are wirelessly relayed to a central decision-making base unit, and actuator nodes, exemplified by an implantable drug delivery device, convert digital signals into biochemical fluxes. Data transferred from the BAN to cloud data handling services can be utilized for direct contact with care providers or emergency services, as well as decision making, analysis, and improved treatment schemes (which can be relayed back to the BAN). The features labeled in black are included in the DNS proof-of-concept while greyed-out features are meant to illustrate the broader vision for the DNS platform. X-ray illustrations adapted from SciePro/Shutterstock with permission.

Here, we report just such a smart healthcare BAN integrating all three components. Health status monitoring (motion sensing) is wirelessly coupled with treatment (neurotransmitter delivery) using organic bioelectronic sensors and actuators (Fig. 1) operating on the scale of 1 Hz (sensing-to-delivery time, Supplemental Video 1) and controlled by machine learning algorithms. The system is a proof-of-concept whereby sensor information can be securely relayed to a decision-making hub (by encrypted/authenticated protocols) where appropriate actions can be initiated based on instantaneous need, e.g., contact physician, administer treatment, alter drug dosage, or initiate other auto-responses. Wireless communication of data between separate nodes is achieved using the human body itself as the data transmission pathway and enhancing security by obviating the need for broadcasting over radio. Data can additionally be transmitted outside the BAN from a central unit to a cloud data handling service for further processing, analysis, and improved prediction models, thereby moving towards continuously optimized and autonomous treatment schemes. To illustrate the concept, we created a functioning web from wearable nodes of sensors and actuators (chemical delivery devices) joined through the capacitive field of the human body itself. The network nodes were seamlessly integrated using IoT-compatible solutions such as low-cost embedded systems (e.g., open-source Arduino platforms) and custom-made solutions. The system unifies the advances in healthcare technologies described in the previous paragraph into a platform resembling the self-regulatory properties of the nervous system, i.e., the ability to sense, communicate, decide, and react accordingly. In this sense the smart healthcare BAN operates as digital nervous system (DNS, Table 1) with the potential to augment the biological nervous system and even supplant dysfunctional/diseased nervous system components.

**Table 1:** Comparison of the digital nervous system (DNS) to the biological nervous system.

Component	Attribute	Biological nervous system	Digital nervous system (DNS)
Physical sensor	Input signal	Muscle extension/contraction	Muscle to sensor extension/contraction
	Signal transducer	Sensory nerve	Stretchable sensor
	Output signal	Neurotransmitter, action potential	Change in capacitance
Biochemical regulation node	Input/trigger signal	Action potential, neurotransmitter	Wireless electronic signal
	Biochemical release component	Axon terminal, synapse	Organic electronic ion pump (OEIP)
	Output signal	Neurotransmitter	Neurotransmitter, neuro-active compound
Intra-body communication	Signal	Action potentials	Wireless electronic signals
	Signal transporter	Neurons, nerve bundles	Body-coupled communication system (BodyCom™)
Processing and decision making	Central processing unit	Central nervous system	Cloud data handling system (Hopsworks)
	Intelligence	Biological neural network (brain, spinal cord)	Deep-learning algorithms

## Results

Neurological disorders represent a particularly challenging use case scenario for IoT in healthcare. To combat the effects of these disorders, such as epileptic seizures or Parkinsonian tremors, there is a need for many distributed sensor signals as well as specifically-targeted and personalized drug delivery. The highly individual nature of indicators, such as respiration, motion, and tremors, and other detectable changes prior to a disease event (e.g. seizure) implies that personalized solutions would have an immense impact on early prevention^22^. For these reasons, we chose a significantly simplified model of a movement disorder to demonstrate the DNS: movement was exemplified by motion/gestures and therapy was exemplified by highly localized chemical delivery.

**Figure 2:**
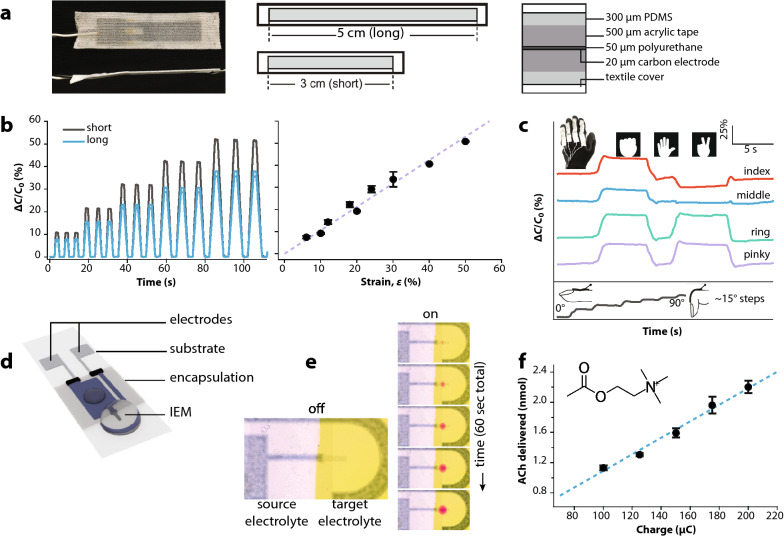
Sensor and actuator/delivery nodes. (**a**) Capacitive strain sensors (short and long types) comprised of a polyurethane dielectric sandwiched by stretchable carbon electrodes encased in rubbers and textiles. (**b**) Relative change in capacitance (Δ*C*/*C*_0_) under repetitive cycling and increased strains, and the extracted linear response with strain up to 50% (2 devices of each length used/shown). (**c**) Signals from gesture capture glove with four individual finger sensors during a game of rock-paper-scissors and for incremental finger bending movements. (**d**) Illustration of an organic electronic ion pump (OEIP) delivery device comprised chiefly of electrodes and an ion exchange membrane (IEM), encapsulated on a flexible or rigid substrate. (**e**) Visualization of ion delivery by transportation of H^+^ from a source to a target electrolyte containing a pH indicator. The proton gradient increased and diffused radially away from the outlet during operation (3 V, on for 1 min). (**f**) Delivery of the neurotransmitter acetylcholine (ACh^+^, chemical structure inset) controlled by a portable driver unit (measuring charge as integrated current) compared to measured ACh^+^ amount.

The sensory input comprised wearable sensors incorporated into textiles that were able to capture human motion. We chose to use wearable strain sensors, as this type of sensor has been shown previously as a viable system for capturing such motion^23^. The wearable strain sensors chosen—based on mechanical durability and robustness—were comprised of stretchable carbon electrodes separated by a dielectric (polyurethane, PU), all encased by acrylic tape and polydimethylsiloxane (PDMS) layers and a textile cover (Fig. 2a)^24^. The sensors exhibit variation in capacitance depending on geometrical changes with low hysteresis characteristics. In brief, as the sensor is stretched along its long axis, the width and thickness are reduced in accordance with the Poisson effect resulting in an overall increased capacitance. Assuming the elastomer behaves as an isotropic and incompressible material, the capacitance follows the ideal parallel-plate model with a linear relationship between capacitance and strain. This model predicts a capacitive gauge factor of 1, defined as (Δ*C*/*C*_0_)/*ε* where Δ*C* denotes change in capacitance, *C*_0_ initial state capacitance, and *ε* strain. We investigated the samples’ sensitivity and stretchable capabilities by cycling the sensors at a deformation rate of 5 mm/s with increasing strains. Deformation and relative capacitive changes of sensors of two different lengths, short and long (30 and 50 mm, both 10 mm wide), showed excellent linearity with strain ranging from 0 to 50% and a capacitive gauge factor of 1.0 (r^2^ = 0.98, Fig. 2b). The sensors maintain their sensitivity for strains up to 50% and exhibit high durability (1000 cycles between 20 and 40% and 30–50% strain repeated twice, Figs. S1, S2). The capacitive sensors exhibit low hysteresis behaviour as quantitatively assessed by calculations of the degree of hysteresis according to previously established methods (Fig. S3 and Table S1)^25^, i.e., comparing the repetitive loading and unloading of the strain sensor. For 1000 cycles, experiencing the same window of applied strain, the recorded output data between the 5th to 95th percentile falls within a relative capacitance range less than 3%.

The goal was to integrate wearable sensor nodes to detect physiologically relevant body motion and differentiate between symbolic healthy and unhealthy states, using simplified model systems in place of more advanced healthcare applications such as detecting epileptic seizures. These model systems enabled us to determine the healthcare BAN’s overall abilities and to ascertain the advantages afforded by implementing deep learning on the sensor signals. The first model system to demonstrate monitoring of health parameters comprised a strain sensor fixed to a flexible belt worn across the chest or abdomen to monitor respiration via readouts using an Arduino platform. Sensors placed in both positions recorded distinct magnitude and frequency variations between inhalation and exhalation, and normal and deep breathing (Fig. S4, relative capacitive changes ranging between ~ 5–10% and ~ 25–30%, respectively). In contrast, abnormal breathing, such as shallow/chest breathing, was denoted by a lack of motion response (relative capacitive changes < 1%) in sensors placed on the abdomen, illustrating how multiple positions or separate sensing nodes can be used to monitor and distinguish various basic physiological patterns. An additional model system for monitoring distinguishable body movements and patterns is gesticulation, i.e., hand or finger movement (Fig. 2c). We thus manufactured a gesture capture glove with four wearable sensors attached to the index, middle, ring, and pinky fingers. Again, the sensor response was recorded via an Arduino. In this application, the sensors were strained upon finger bending resulting in relative capacitive changes from 0 to ~ 25% when starting from an open position and proceeding to a closed palm position. From this it is easy to distinguish various gestures in real time, as exemplified by the familiar rock-paper-scissor hand game (Fig. 2c). In future smart wearables, the movement repertoire can of course be expanded and fine-tuned by incorporating more sensor nodes. For example, as the sensors are capable of accurately detecting even small displacements and motions, such dynamic ∆*C*/*C*_0_ patterns would be capable of detecting even small displacements or other abnormal motion patterns.

The organic electronic ion pump (OEIP)^26,27^ is an iontronic/electrophoretic delivery device exhibiting high electronic and pharmaceutical dose precision and established autoregulation capabilities when coupled to sensors^28,29^. For instance, electronic control over the delivery of the neurotransmitter γ-aminobutyric acid (GABA) resulted in highly localized suppression of epileptiform activity in rodent hippocampal slice models^29^. OEIPs are an ideal drug delivery node for the DNS because they are electronically addressable, can accurately translate electronic signals into precise delivery of (charged) biochemical substances (Fig. 2d–f), and have been demonstrated to regulate and provide therapy for neurological disorders including pain^30^ and epilepsy^29^. In OEIPs, the number of ions delivered is directly proportional to the time-integrated current (i.e., total charge) passed through the circuit. Based on this principle we manufactured a custom-made portable/wearable OEIP driver with current integrator circuitry. When activated, the unit supplied a constant voltage (0–5 V) to the OEIPs for a set amount of time or set quantity of charge delivered. The multipurpose and adaptable driver solution allowed operation of a wide range of OEIPs, which is important as pump designs and their requirements (voltage, delivery dynamics, etc.) vary depending on the application. The operation and control of OEIPs were tested using the custom driver as well as traditional source-meter units. OEIPs were either microfabricated by photolithographical patterning techniques^31^ or screen-printed^32^ as previously established.

OEIPs with a relatively short and wide channel (1–2 mm and 200–500 µm, respectively) exhibited the most noticeable visual response of actuation. The on-state of a pump (applied voltage of 3 V) was displayed through transportation of protons (H^+^) from the source reservoir containing 10 mM HCl(aq) to the target electrolyte containing a pH indicator (Fig. 2e; Supplementary Movie 1). This resulted in H^+^ gradients and nearly instant color change from yellow to red at the outlet, a pattern which diffused radially outward over time. Next, the controller circuit’s capabilities were tested by driving the OEIP at 4 V to transport the neurotransmitter acetylcholine (ACh^+^) from the source to the target. The charge limit, i.e., time-integrated current, was set over the range 100–200 µC and compared to the actual amount of delivered ions in the target solution, quantified using a fluorometric assay. Figure 2f shows an excellent linear correlation between the set and measured amount of ACh^+^, revealing that the driver enabled direct control over delivery via determined charge limits.

A body-coupled communication (BCC) system (BodyCom system, Microchip Technology Inc.) was explored as a secure and energy-efficient BAN communication pathway. Our modified version of the system allowed for transmission of data between separate sensor nodes located at several locations on the body, granted that they were in close proximity (touching or within a few centimetres) to the same human body skin^33^. This custom setup thus offers a user-friendly secure connection between the sensing elements, which inform healthcare decisions, and actuating devices, which carry out appropriate therapies (e.g., delivery of relevant neurotransmitters). The electrical signal is propagated along the surface of the skin by means of a capacitive field as the communication pathway to transfer short messages, sensor/actuator data, keys, or identity information between BCC mobile tags and a base unit (Fig. S5). For this purpose, sensor responses were collected, locally processed (via Arduino) and translated into short messages, e.g., representing different states such as “one finger bent”, “open hand”, or even raw sensor data. The overall BCC systems used in our experiments consists of a base unit, provided by Microchip but with modified software, and a BCC mobile unit, usually referred to as the “BCC tag”, which we have designed and manufactured. In a typical setup there is one base unit managing communication between several mobile units using the capacitive coupling technique. The base unit continuously requested and received sensor data as well as transmitted this information to the actuator BCC tag for decision-making to trigger the corresponding action.

**Figure 3:**
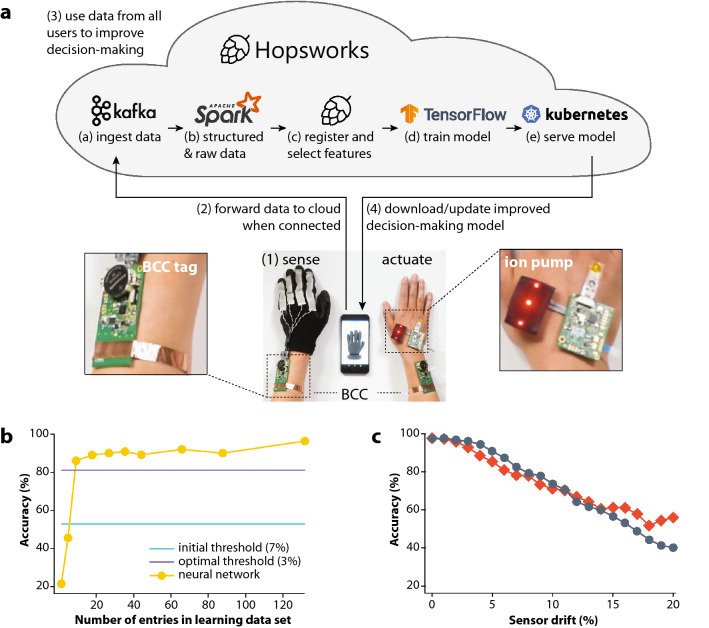
Digital nervous system integration and implementation of machine learning. (**a**) The DNS proof-of-concept, where the sensing node (glove) state was received by the base unit/tag (in contact with person, but out-of-view during filming) and forwarded to the actuator node (ion pump) through the capacitive field of the body. The received state was further sent from the base unit to the mobile device via Bluetooth for interaction with the Hopsworks cloud-based machine learning system (steps 1–4 and **a**–**e**). (**b**) Accuracy of a neural network classification of the hand position as a function of the quantity of data used to train it, compared to the accuracy of an initial estimated threshold and an optimized threshold corresponding to bent fingers. (**c**) Accuracy of a neural network classification of the hand position as a function of the simulated drift in sensor measurements compared to the training data. The drift was applied on one finger (grey) or all fingers (orange) at the same magnitude. Photographs in part a taken by Thor Balkhed at Linköping University, and used with permission.

Additionally, data collected by the base unit was communicated to external mobile devices via Bluetooth. The sensor and actuator data could thus be forwarded to a dedicated cloud data handling service for storage, analysis, training of machine learning models, and eventually prediction via these models for improved detection limits and therapies (Fig. 3a). Here, a Hopsworks^34^ cloud platform was utilized and further developed to streamline the flow and handing of large e-health data, as more nodes can be coupled to the BAN system to increase available health monitoring parameters. Data gathered from individual users, or from all users, can be fed into the platform to optimize the decision-making model and prediction accuracy of the neural network and improved models can thereafter be downloaded from the cloud to the BAN nodes. An instructive experiment was carried out in which 16 willing individuals wore the motion-capture glove while repetitively cycling through a determined list of five possible gestures. Notably, the individual users’ bending response ranges and patterns varied, partially due to the fit of the glove (Fig. S6). The collected data was then used to train a two-layer classification network implemented in TensorFlow. After running the hyperparameter exploration, an accuracy of 97% was reached (Fig. 3b). In contrast, our first solution to predict the hand position, i.e., setting a stretch threshold of 7% to correspond to a finger bending, had an accuracy of 53% and finding an optimal threshold (3%) with hindsight only provided an accuracy of 82%. To illustrate the advantage of continuously gathering data and training, we measured the evolution of the accuracy with respect to the quantity of data used for the training (Fig. 3b). This scenario was rather simple, leading to a rapid increase in accuracy when modelled despite variations in how well the glove fit the participant and associated variations in strain recordings depending on hand size. However, considerably more complex and medically relevant questions, *e.g.,* epileptic seizure prediction, will require larger data sets and distribution of sensors.

In addition to improved accuracy compared to empirically chosen decision-making thresholds for sensor data, deep learning provides robustness. To demonstrate this, two separate experiments were carried out: a sensor drift simulation (e.g., due sensor degradation/aging) and an ablation test (e.g., complete loss of one of the sensor signals). The simulation showed that the deep learning system can handle up to 3% drift without losing less than one point of accuracy (Fig. 3c). The loss in accuracy thereafter increases linearly with higher drift. In comparison, applying a drift larger than 3% for a threshold-based system (with a threshold of 3%) rapidly results in accuracy of 0. Moreover, continuously sending data to Hopsworks would allow the system to detect the drift and to train the deep learning system to adapt accordingly. The accuracy after training with drift taken into account was 97%. With a threshold-based approach the ablation of one sensor results in the impossibility of differentiating between two hand positions. The accuracy is at best 80% and software correction is not possible. With the deep learning system, the accuracy also falls under 80%. However, retraining the system allows rapid detection of the absence of one of the sensors, and after the retraining an accuracy of 89% is obtained. This is because the bending of one finger also modifies the position of the other fingers and the deep learning system can detect and use even slight finger position modifications to improve the accuracy. This highlights the importance of having multiple sensors measuring different metrics for both increased accuracy and redundancy. It also becomes apparent that the deep learning system can detect and learn from complex and subtle patterns, as well as handle sensor failure thanks to its capacity to identify hidden patterns.

**Figure 4:**
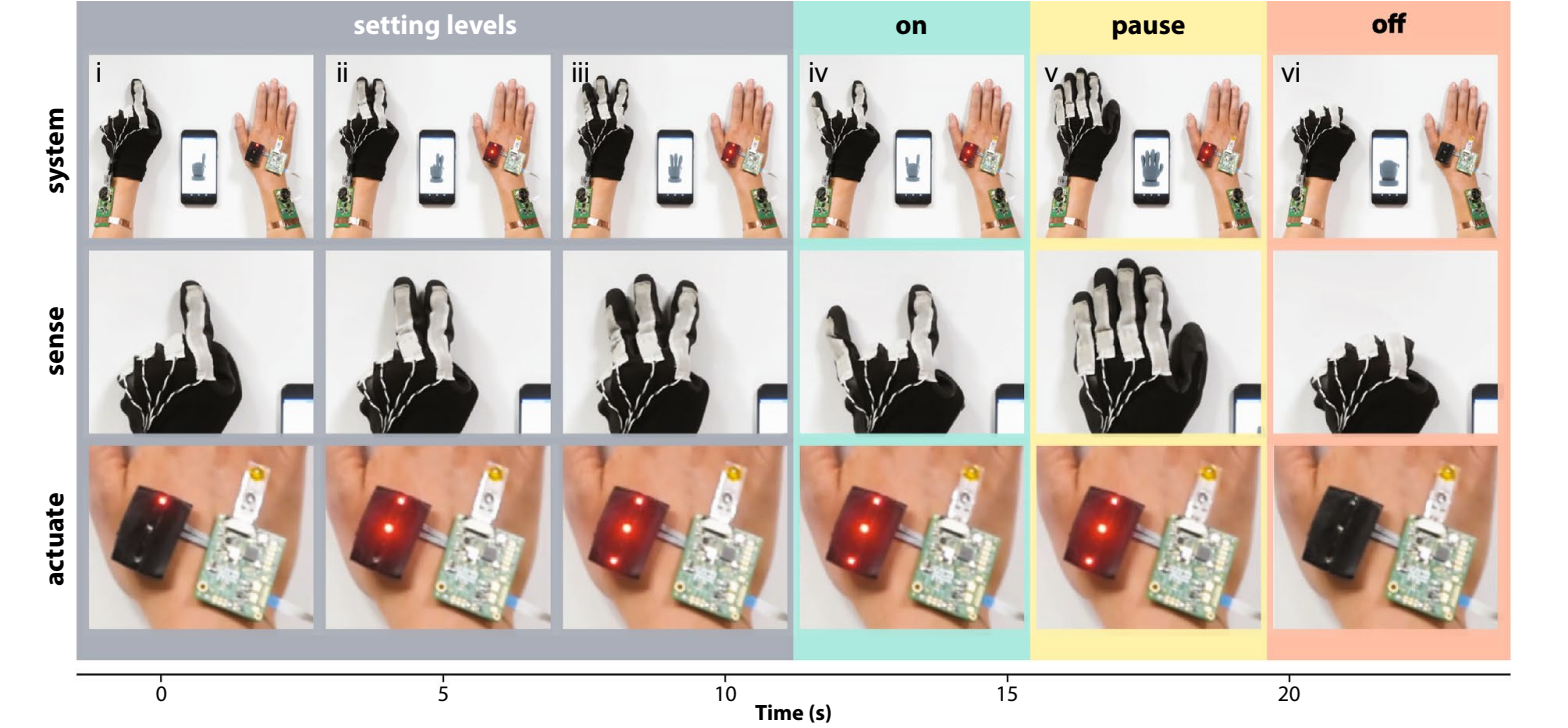
Demonstration of the digital nervous system (DNS) proof-of-concept. Time-lapse and corresponding image panels (system overview, sense and actuator nodes; Supplementary Movie 1) displaying: setting the drug dispensing levels by counting to three (**i**–**iii**), pump activation by ‘rocker’ gesture (**iv**), pausing (**v**), and clearing the system (vi) and associated LED array responses next to an inserted ion pump. Photographs taken by Thor Balkhed at Linköping University, and used with permission.

The DNS proof-of-concept was achieved by bringing together all the components in a fully functional wireless closed loop system, as visualized in Fig. 3a (Supplementary Movie 1). The sensor node comprised the gesture glove, Arduino controller, and BCC-tag. The actuator node comprised the OEIP, Arduino controller, BCC-tag, and additional indicator LEDs. Gestures corresponded to different states that each triggered a specific action within the BAN, as visualized in Fig. 4. Specifically, counting on one, two, or three fingers (Fig. 4i–iii) corresponded to setting three drug delivery levels (charge limits of 100, 150, or 200 µC, respectively); the “rocker” gesture (Fig. 4iv) signalled activation of the pump (on-state until the specified amount had been delivered); open palm paused the action (Fig. 4v); and closed fist (Fig. 4vi) cleared all commands and brought the system back to its default off-state. The base unit continuously requests the status from all mobile tags coupled to the body (Supplementary Movie 2). Upon a change of the state in the glove multi-sensor node, the base unit pushed the state to the actuator BCC tag (in real-time), while simultaneously sending the information to a mobile device via Bluetooth to visually confirm the state alteration and to relay information to the cloud for machine learning and progressively improved decision-making. During the visual confirmation, a delay between the hand gesture and display on the mobile device was noted (Supplementary Movie 1), which largely stemmed from a non-optimized Bluetooth connection between the BCC base unit and mobile device. As can also been seen (Supplementary Movie 1), there was no delay between the hand gesture and activation at the actuator tag (OEIP). Once the actuator tag receives a specific state, it transmits the corresponding decision for action to the controller unit to engage the OEIP according to the previously described protocols. OEIP operation was visually aided by an array of three LEDs, where setting of three drug delivery levels was represented by turning on one, two, or three LEDs (indicating 100, 150, or 200 µC, Fig. 4i–iii); the on-state was displayed by blinking (Fig. 4iv); and all LEDs were turned off as the system was cleared (Fig. 4vi). As soon as a given pumping sequence was completed, the status of the actuator node changed and was detected upon the next status request by the base unit and thereafter relayed to the mobile device via Bluetooth. Additionally, all data received by the mobile device could be uploaded to the cloud service. The stored sensor and actuator data can thereby be accessed by the user or their physician, and further evaluated to improve detection and treatment schemes.

## Discussion

A key-component of future digital healthcare is a complete patient-adapted system for diagnosing and decision-making, which initiates optimal protocols to suppress symptoms and therapy. It should be grounded on reliably generated data, safe transfer and communication of information, accurate and swift decision-making, and personalized and precise delivery of treatments. Here, we have chosen two model systems (breath, gesticulation) of highly personalized information to determine the DNS’s capabilities and ascertain if deep learning can be used to increase the robustness and utility of the system, making the leap from wellness applications (e.g., wearable fitness trackers) to true healthcare applications feasible. Therapy for neurological disorders is the ultimate goal for the DNS. We thus aim to develop and demonstrate an integrated and complete digital healthcare system recording relevant physical parameters to dictate the delivery of neurotransmitters, *e.g.*, for supressing epileptiform activity^22^.

After embracing these challenging criteria, we developed a comprehensive organic–inorganic sensor-actuator BAN, coupled to cloud-based deep learning. The resulting system comprises sensing and recording of body motion, parallel transmission of data to the cloud for neural network training and via body-coupled communication to electronically drive the delivery of biochemical substances (the neurotransmitter acetylcholine). This is achieved on the scale of 1 Hz (Fig. 2d–f, Fig. 4, Supplementary Movie 1) which is within the temporal range of neurological disorders and symptoms such as pain^35^, epilepsy^22^, and tremor^36^. In future embodiments of the system, advances in high-speed OEIP technology (on the scale of 1–10 ms switching^37,38^) could greatly enhance temporal dynamics.

In this demonstration of a digital nervous system we have included organic bioelectronic sensor and actuator nodes, owing to their desirable characteristics with respect to flexibility, stability, and biocompatibility, and with the focus on large-area wearable sensors and implantable electronic-to-biochemical actuation. One leg of the parallel communication pathways includes low-power body-coupled communication built up from inorganic (Si, metal) electronics, which is based on capacitive coupling operating at 125 kHz, providing secure transmission of low bitrates confined to the body. The other is based on Bluetooth, connectivity to the cloud and deep learning, the latter performed using the scalable Hopsworks cloud platform which is optimal for large data and multi-node systems. These two legs of the system also exhibit different timescales and thus different modes of application in e-health settings. The faster BAN (without relying on the cloud) would be ideal for rapid decision making such as treating an oncoming epileptic seizure. The cloud-enabled component, relying on local internet connectivity and latency to the nearest data center, would be more ideal for updating on-body algorithms/models or for much less time-sensitive decision making, such as adjusting dosages over the course of hours or days. In terms of power needs, the total peak power consumption is around 250 mW in the full setup. However, the most power-hungry components, e.g., Bluetooth and excessive amount of interfacing circuits, can be reduced. The system can be refined by putting more devices into sleep mode, migrating to the latest BLE standard, or reducing the complexity of the BCC base board. We find that the power can be reduced by a factor of almost 15 down to below 20 mW (almost 150 h with a coin cell battery) by optimizing the discrete components. Power consumption can be further reduced by integrating more functionality in dedicated ASICs. By strategically marrying organic and inorganic systems, we were able to harness the unique features of organic electronics and the computing/communication power of inorganic systems^39^.

However, this integration also exposes a number of challenges, which provide crucial input for further development within relevant research fields. Using machine learning, and particularly deep learning, in healthcare benefits early disease detection and patient care^11^ but it requires a significant amount of training data. Reliable wearable sensors that provide operational and long-term stability, with suitable output characteristics that empower easy implementation into digital communication and processing, are therefore crucial. With body-coupled communication, low operational and standby power is yet another important factor, suggesting the use and further development of organic sensors and actuators that consume minimal amounts of energy while in drift or at rest, as well as self-powered concepts^40,41^. Another challenge relates to the narrow bandwidth of body-coupled communication, which results in an increased probability for data packet collisions as the number of pathways and nodes increases. Furthermore, connectivity to conventional complementary metal–oxide–semiconductor (CMOS) technology becomes challenging since the number of contact pads on the CMOS device drives cost. With multiplexers and demultiplexers, realised in printed organic electronics, fewer contact pads on lower-cost silicon chips can then handle a very large number of peripheral wearable sensor and actuator nodes. This then goes hand in hand with our results that clearly show that multiple sensors and accurate sensing facilitate robust deep learning for decision making and reduce the need for frequent retraining. To enable such complexity, while simultaneously miniaturizing individual nodes, custom-made application-specific integrated circuit application-specific integrated circuits (ASICs) can be implemented. ASICs would replace bulky circuit boards and generic controllers. Once the substantial ASIC development cost is overcome, the cost-per-unit could greatly reduce the cost of integrated healthcare systems as we demonstrate here. Finally, security, integrity, and reliability of connected wearable IoT technology is yet another challenge indicating the importance of developing and implementing tailor-made protocols for encryption and identification keys that ensure immunity to eavesdropping or interference of sensitive information and eventual therapies.

## Methods

### Sensor node

#### Strain sensor fabrication

Strain sensors (C-Stretch) were manufactured by Bando Kagaku. Sensors of two different lengths were utilized (10 mm wide, and 30 or 50 mm long for short and long sensors, respectively). The sensors are designed to be longer in the strain direction and are thus more sensitive to strain in the “intended” (long) direction. In terms of sensitivity, they exhibit a linear capacitance response up to 200% and limit of detection 10% (or lower)^42^.

#### Characterization

A linear stretcher equipped with a stepper motor controlled by a custom-made LabVIEW program was used to apply tensile strains. The sensors were fixed to the stage and repetitively loaded and unloaded with strains ranging from 0 to 50% increasing stepwise. The durability and stability were further assessed by cycling 1000 times between 20–40% and 30–50% strain (repeated twice). Sensors were fixed to a stretchable belt and glove for breathing and gesture capturing experiments. The sensors were coupled to an Arduino Uno or Pro Mini microcontroller board (ATmega328P) either directly or wirelessly (Arduino Bluetooth unit) connected to a computer for data acquisition of capacitive responses during stretching and on-body tests.

### Communication nodes

#### Body-coupled communication

The body-coupled communication system (BodyCom) base board development kit was acquired from Microchip Technology Inc. The development board makes use of capacitive coupling to communicate between sensors in or around the vicinity of the human body. Both base and mobile units were equipped with a touch sensor and a coupling pad as a means of contact to the human subject. BCC mobile-tags were re-designed to allow access to the microcontroller’s serial communication functionality such that they could easily communicate with the sensor and actuator nodes. The BCC units communicate at a default low frequency of 125 kHz, which does reduce the bandwidth, but offers a more secure communication as the signal will not radiate as much. The system can be made robust against interference from other sensors as the base board decides by polling which BCC mobile tag should be allowed a communication slot. Additional information on the BodyCom is found in the Supplementary Material.

### Cloud service and security

Hopsworks is a secure big data multitenant platform, relying on the concept of projects to ensure its security and multitenancy. A project is a set of users and datasets. Each user of the platform is given a different identity (project user) for each of the projects they are part of, guaranteeing that a user cannot cross reference data from one project to the other. Datasets can be shared between projects only if the project administrator allows it. An example of utilization would be to have a project for a patient with the patient and their doctor as member of the project. They could then decide to share some of the project datasets with a deep learning project to improve the accuracy of the patient treatment. Thanks to the project isolation the data, scientists working on the deep learning project are not able to compare the data of this project to data they may have access to in other projects, thus ensuring privacy of the patient. Project users are securely identified using X.509 certificates and JSON Web Tokens (JWTs). The certificates are used inside Hopsworks to identify the project user and to encrypt communication between machines (TLS). JWT tokens are used for the user to connect to Hopsworks. For the mobile phone to connect and send data to the cloud, the user needs to first download their JWT token on their phone. They then use this token to establish the connection with the cloud, and the communication between the phone and Hopsworks is secured using the HTTPS protocol.

### Decision-making and machine learning

#### Deep learning

Machine-learning algorithms are used to make predictions about events and trends of interest in the patient sensor readings. Sensor data flows from mobile phones to Hopsworks, and decisions can be taken at any point along the way. Time-critical decisions can be taken at the mobile phone, where embedded models, built e.g., with TensorFlow lite, can be used to identify critical events and notify the patients or systems immediately. The sensor data, used to train these models, is forwarded to Hopsworks that collects and archives all sensor data in HopsFS^43^, that can scale to store hundreds of petabytes. Decisions can also be taken in stream-processing applications deployed Hopsworks. These applications receive streams of sensor data from the mobile phones and can use higher quality models (that are too big to fit on a mobile phone), and further take actions at the level of groups—for example, it could notify groups of patients if an event is of relevance to the group as a whole. As Hopsworks contains the archived sensor data, it is also the place where machine learning models are trained, either on GPUs for deep learning or CPUs for classical approaches, such as decision trees and k-means clustering.

### Actuator node

#### Device manufacture

Organic electronic ion pumps were manufactured by either standard photolithography methods or via screen-printing in accordance with previously reported procedures^31^.

#### OEIP control and testing

Ion pumps were controlled and tested using a source meter (Keithley) or a custom-made driver (combined voltage supply and integrator). Chemicals and solvents were purchased from Sigma Aldrich unless otherwise specified. For visualization experiments, 10 mM HCl(aq) was pumped to a target electrolyte containing a pH indicator (2.5 mM methyl red in 100 mM KCl(aq)). OEIPs were filmed for ~ 3 min upon applying a voltage of 3 V and monitored for 5 min prior to pump activation. In experiments to assess dosage control for the OEIPs, 25 µl acetylcholine chloride (10 mM, aq) and KCl (10 mM, aq) were placed in the source and target reservoirs. The controller was used to apply a potential of 4 V until a determined charge limit was reached. The 25 µl target solution was collected and 95 µl of KCl solution was placed on the target, mixed and added to the collected target aliquot to ensure all delivered acetylcholine was collected. The concentration of acetylcholine in the collected samples was measured via an Amplex Red acetylcholine/acetylcholinesterase assay kit (A12217, Molecular Probes) using a plate reader (Synergy H1, BioTek). The delivered acetylcholine amounts from three devices were measured in duplicates, averaged, and compared to the respective charge limits.

### Human participants

Experiments involving glove training and breath monitoring involved entirely CE-marked components in contact with the body. Experiments involving the full demonstrator system involved non-invasive wearable components placed in short-term contact with skin. All results were obtained from volunteers. The breath sensing experiments were tested on two people (both co-authors). The hand-gesture training utilized 16 volunteers from the Linköping University and RISE teams. The sole participant for the full demonstrator gave written informed consent. All experiments were undertaken following Swedish state and local legislation as well as Linköping University instructions for laboratory work according to the university’s Laboratory Safety Manual (https://insidan.liu.se/miljo/laboratoriesakerhetshandboken/?l=en). All experimental protocols were carried out according to these regulations and approved by authority of Linköping University’s Department of Science and Technology which determined that ethics committee approval was not necessary.

## Supplementary Information

Supplementary Information 1.

Supplementary Video 1.

Supplementary Video 2.
