# Supplementary material for: A digital nervous system aiming toward personalized IoT healthcare
Source: Sci Rep. 2021 Apr 8;11:7757. doi: 10.1038/s41598-021-87177-z (PMC8032817; doi:10.1038/s41598-021-87177-z)
Supplement: Supplementary file 1 — Supplementary Information 1. [file 41598_2021_87177_MOESM1_ESM.pdf]

Supplementary information

A digital nervous system aiming toward personalized IoT healthcare

Astrid Armgarth, Sandra Pantzare, Patrik Arven, Roman Lassnig, Hiroaki Jinno, Erik Gabrielsson, Yonatan Kifle, Dennis Cherian, Theresia Arbring Sjöström, Gautier Berthou, Jim Dowling, Takao Someya, J. Jacob Wikner, Göran Gustafsson, Daniel T. Simon, Magnus Berggren

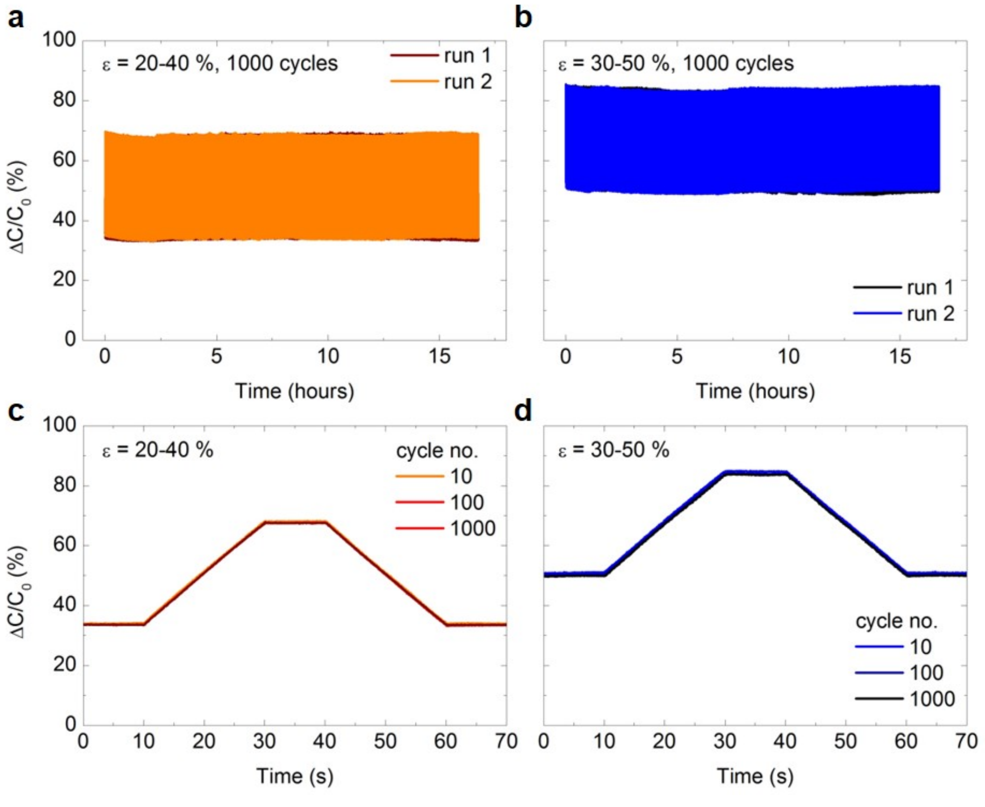

Fig. S1. Sensor durability. Relative capacitance strain sensor responses during cycling between (**A**) 20-40% and (**B**) 30-50% strain, each experiment repeated twice. Extracted individual cycles (10, 100, and 1000) for (**C**) 20-40% and (**D**) 30-50% strain.

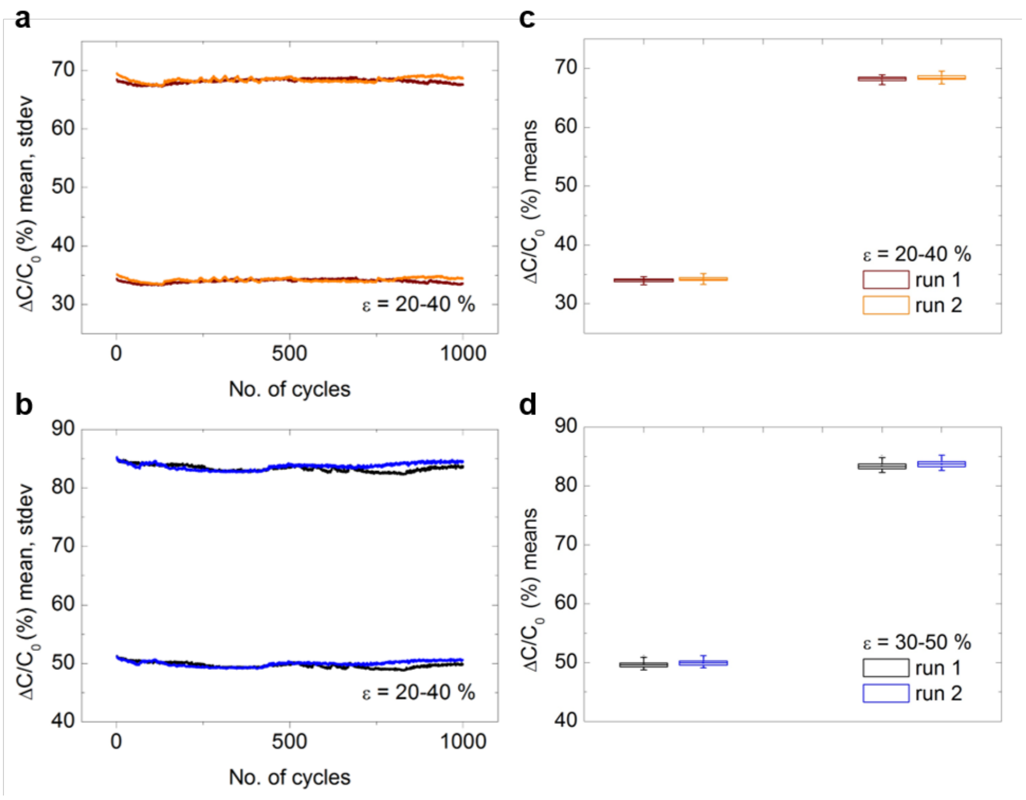

Fig. S2. Sensor stability. Calculated mean relative capacitance strain sensor responses and standard deviations (stdev) over 1000 cycles between (**A**) 20-40% and (**B**) 30-50% strain. (**C-D**) Their corresponding boxplots depicting the median (line), upper and lower quartile (box) and 1.5x interquartile range (whiskers). Each experiment was repeated twice.

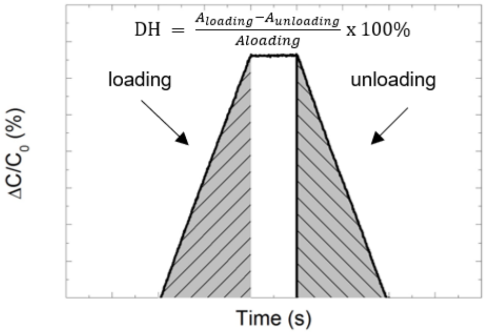

Fig. S3. Degree of hysteresis. Schematic of degree of hysteresis (DH) estimations during loading and unloading of sensors during cycling, according to previously established methods*. Low degree of hysteresis is indicated by a DH value close to 0%.

* SG Yoon, H-J Koo, and ST Chang. *ACS Appl. Mater. Interfaces* **7**, 27562–27570 (2015). doi:10.1021/acsami.5b08404

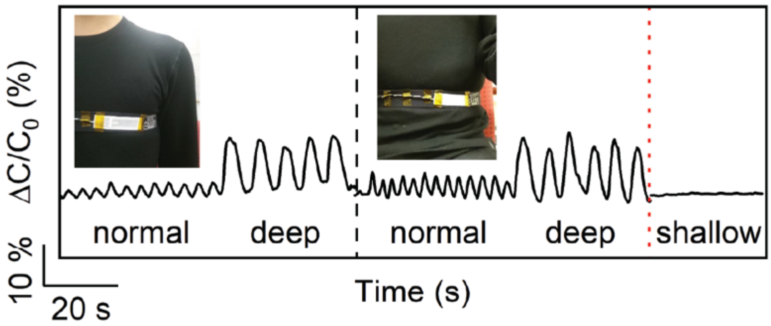

Fig. S4. Capacitive strain sensors fixed onto a belt to monitor normal and deep breathing patterns on chest or stomach positions.

Table S1. Degree of hysteresis. Calculated mean degree of hysteresis from 1000 cycles between 20-40% or 30-50% strain.

**ε (%) cycling range**	**run**	**DH mean (%)**
20-40	1	0.94 ± 0.52
20-40	2	0.86 ± 0.54
30-50	1	1.34 ± 0.57
30-50	2	1.38 ± 0.53

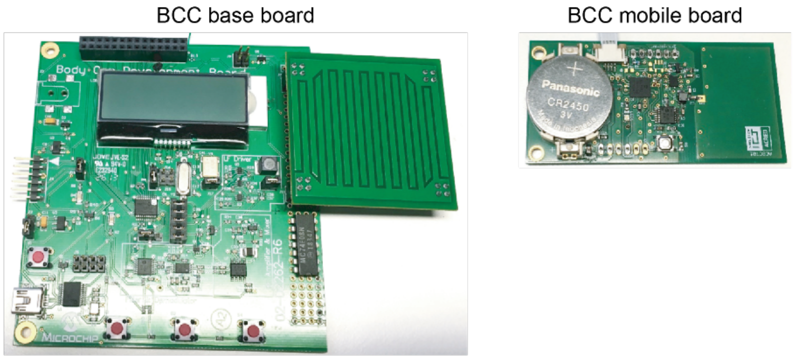

Fig. S5. Body Coupled Communication. Examples of body coupled communication (BCC) base unit and mobile tags. To the left, the evaluation board provided by the manufacturer but with new software and to the right a custom tag developed for increased functionality.

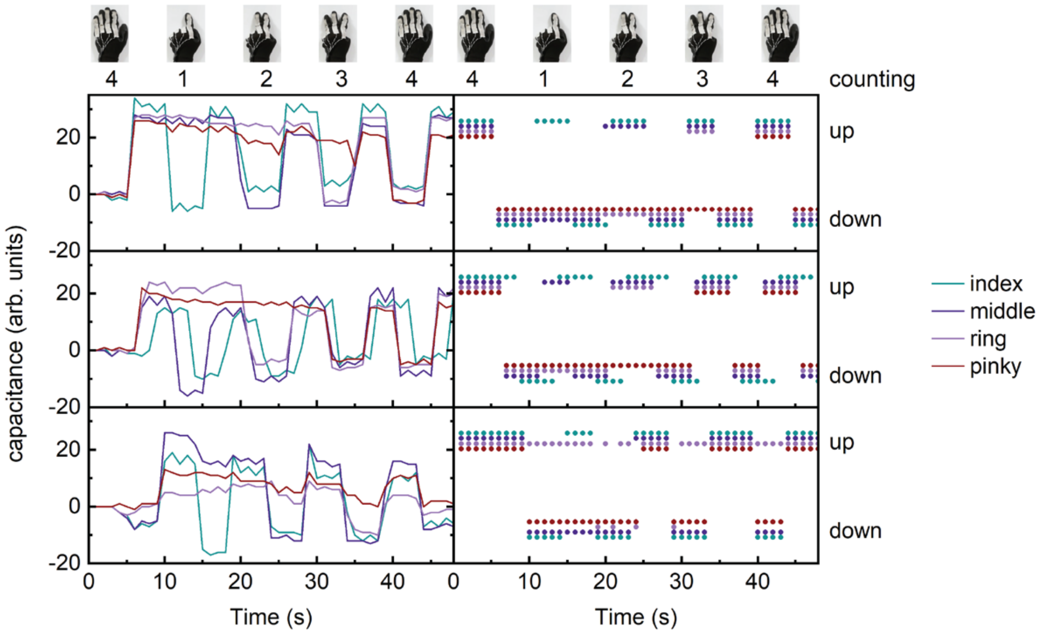

Fig. S6. Sensor data. Data from three participants wearing the gesture capture glove and performing repetitive motions of counting on one to four fingers while making a closed fist in between each movement. An optimized threshold based on the results from the complete study group was used to determine if a finger was up or down (*i.e.*, opened or closed).

Comments on the MicroChip BodyCom kit used in the experiments

The BCC system uses the base components of the BodyCom kit defined by Microchip (https://www.microchip.com/design-centers/embedded-security/technology/bodycom-trade-technology, May 2019) but has since been redesigned both from a hardware and software perspective. The system works in the following manner: there is a base unit connected to the body, and a set of "tags"/slave units. A tag could contain either a sensor or an actuator. The base unit is considered the gateway of the body. In our experiments we only used one.

The base unit can be set to poll tags for response by individually calling them using their unique IDs. Only tags with the polled ID will respond/listen to the commands sent to the base unit allowing for no interference between the units. Time-out functions are implemented to abort waiting for lack of response from malfunctioning tags, etc. The number of sensors will be limited, and time delays are not critical. Also broadcast options are possible from the base unit such that all tags can take actions based on the instruction transmitted by the base unit.

The communication frequency of the downlink (from base unit to tag) differs from the uplink (other direction), and thus downlink communication can never interfere. Instructions sent from the phone to the base unit for further transportation to the tags will be queued based on time of arrival. In each tag and base unit, the receiver and transmitter are tuned to their respective frequency bands of 125 kHz and 8 MHz. Microchip's BodyCom kit then uses a PIC16LF1829 20-pin Flash in the base unit and a PIC16LF1827 18-pin Flash MCU device in the tag to control the modulation of the signal in these bands. The de-/modulated signals are further filtered and amplified. In the transmit path, the voltage of an electrode in close proximity of the body is affected, and the variation of the electrical field can be detected by the other unit. The challenge is that the signal is typically heavily attenuated due to the properties of the human as a communication channel and the receivers have to react on very weak signals.

The base unit can also be set in "listen mode" and wait for tags that themselves decide to transmit (through the detection of a user interaction, passing a certain threshold, etc.). In this scenario, there is a potential risk for interference between signals from tags towards the base unit. However, messages are short (their duration is in the order of a few ms) thus unlikely to collide. If there is a collision, due to a cyclic redundancy check (CRC) mechanism, the message will be discarded by the base unit. Upon detection of a corrupted message in "listen mode" the base unit could decide to go back into polling mode and poll all tags for their latest message. In "listen mode" a future system could involve more controlled turn-based communication between tag and base unit where each tag is allocated a certain time slot. The base board can also call for resubmission of an incomprehensive message and allocate random time delays to the sensors forcing them to not submit their responses repeatedly.
